# Identification and Validation of Hub Genes and Construction of miRNA-Gene and Transcription Factor-Gene Networks in Adipogenesis of Mesenchymal Stem Cells

**DOI:** 10.1155/2024/5789593

**Published:** 2024-08-29

**Authors:** Miaomiao Dai, Weisheng Hong, Yi Ouyang

**Affiliations:** ^1^ Department of Ophthalmology Shunde Hospital Southern Medical University (The First People's Hospital of Shunde, Foshan), No. 1 Jiazi Road, Lunjiao, Shunde District, Foshan City, Guangdong Province, China; ^2^ Department of Joint Surgery Shunde Hospital Southern Medical University (The First People's Hospital of Shunde, Foshan), No. 1 Jiazi Road, Lunjiao, Shunde District, Foshan City, Guangdong Province, China

## Abstract

**Background:**

Adipogenic differentiation stands as a crucial pathway in the range of differentiation options for mesenchymal stem cells (MSCs), carrying significant importance in the fields of regenerative medicine and the treatment of conditions such as obesity and osteoporosis. However, the exact mechanisms that control the adipogenic differentiation of MSCs are not yet fully understood.

**Materials and Methods:**

We procured datasets, namely GSE36923, GSE80614, GSE107789, and GSE113253, from the Gene Expression Omnibus database. These datasets enabled us to perform a systematic analysis, including the identification of differentially expressed genes (DEGs) pre- and postadipogenic differentiation in MSCs. Subsequently, we conducted an exhaustive analysis of DEGs common to all four datasets. To gain further insights, we subjected these overlapped DEGs to comprehensive gene ontology enrichment and Kyoto Encyclopedia of Genes and Genomes pathway analyses. Following the construction of protein–protein interaction (PPI) networks, we meticulously identified a cohort of hub genes pivotal to the adipogenic differentiation process and validated them using real-time quantitative polymerase chain reaction. Subsequently, we ventured into the construction of miRNA-gene and TF–gene interaction networks.

**Results:**

Our rigorous analysis revealed a total of 18 upregulated DEGs and 12 downregulated DEGs that consistently appeared across all four datasets. Notably, the peroxisome proliferator-activated receptor signaling pathway, regulation of lipolysis in adipocytes, and the adipocytokine signaling pathway emerged as the top-ranking pathways significantly implicated in the regulation of these DEGs. Subsequent to the construction of the PPI network, we identified and validated 10 key node genes, namely IL6, FABP4, ADIPOQ, LPL, PLIN1, RBP4, ACACB, NT5E, KRT19, and G0S2. Our endeavor to construct miRNA–gene interaction networks led to the discovery of the top 10 pivotal miRNAs, including hsa-mir-27a-3p, hsa-let-7b-5p, hsa-mir-1-3p, hsa-mir-124-3p, hsa-mir-155-5p, hsa-mir-16-5p, hsa-mir-101-3p, hsa-mir-21-3p, hsa-mir-146a-5p, and hsa-mir-148b-3p. Furthermore, the construction of TF–gene interaction networks revealed the top 10 critical TFs: ZNF501, ZNF512, YY1, EZH2, ZFP37, ZNF2, SOX13, MXD3, ELF3, and TFDP1.

**Conclusions:**

In summary, our comprehensive study has successfully unraveled the pivotal hub genes that govern the adipogenesis of MSCs. Moreover, the meticulously constructed miRNA-gene and TF–gene interaction networks are poised to significantly augment our comprehension of the intricacies underlying MSC adipogenic differentiation, thus providing a robust foundation for future advances in regenerative biology.

## 1. Introduction

Mesenchymal stem cells (MSCs), characterized by their pluripotent nature, are widely distributed within various mesenchymal tissues, including but not limited to bone marrow, placenta, adult muscle, umbilical cord blood, dental pulp, and adipose tissue [1, 2, 3]. MSCs exhibit remarkable attributes, such as self-renewal capacity and the potential for multidirectional differentiation, enabling them to differentiate into diverse cell lineages, such as osteocytes, chondrocytes, adipocytes, fibroblasts, skin cells, neural cells, or hepatocytes, contingent upon specific inductive cues [4, 5]. Notably, adipogenic differentiation represents a pivotal avenue in the differentiation repertoire of MSCs, holding substantial significance in the realms of regenerative medicine and addressing conditions like obesity and osteoporosis [6, 7]. Nevertheless, the precise mechanisms governing the adipogenic differentiation of MSCs remain incompletely elucidated.

The comprehensive comprehension of the intricacies underlying adipogenic differentiation holds significant promise, as it could inform the development of more refined seed cell-based strategies rooted in MSCs for regenerative medicine and pave the way for more efficacious treatments for associated pathological conditions. Recent strides in high-throughput sequencing and microarray technologies have presented an opportunity for a systematic and exhaustive exploration of the mechanisms governing adipogenic differentiation [8, 9, 10, 11]. However, the challenge of attaining precise results persists due to the inherent susceptibility of independent microarray analyses to false-positive outcomes.

In the present investigation, our study delved into this challenge by accessing and analyzing four distinct datasets sourced from the Gene Expression Omnibus (GEO) database. This rigorous analysis identified a set of differentially expressed genes (DEGs) that distinguished the control group from the adipocyte induction group. Subsequently, we embarked on elucidating the underlying biological mechanisms through the application of gene ontology (GO) and Kyoto Encyclopedia of Genes and Genomes (KEGG) pathway enrichment analyses. Furthermore, we constructed vital networks, including the protein–protein interaction (PPI) network, miRNA-target gene network, and transcription factor (TF)-target gene network. These comprehensive endeavors serve as a significant step toward shedding light on the intricate molecular machinery governing the adipogenic differentiation of MSCs.

## 2. Materials and Methods

### 2.1. Data Acquisition

The GEO repository (http://www.ncbi.nlm.nih.gov/geo) served as the primary source for a comprehensive compilation of microarray, next-generation sequencing, and other high-throughput functional genomic datasets. Specifically, four distinct datasets, namely GSE36923 [8], GSE80614 [9], GSE107789 [10], and GSE113253 [11] were meticulously retrieved from the GEO database. These datasets encompassed adipogenically induced human bone marrow-derived mesenchymal stem cell (hBMSC) samples, and their salient details have been thoughtfully cataloged in Table 1.

### 2.2. DEG Identification

To identify DEGs within the aforementioned datasets, we harnessed the capabilities of Networkanalyst (https://www.networkanalyst.ca/NetworkAnalyst/), a robust online platform engineered for the comprehensive analysis of transcriptome profiles, meta-analysis, and systems-level interpretation of gene expression data [12]. Following meticulous data normalization, DEGs were discerned, employing stringent criteria, namely |log FC| ≥ 1 and *P* < 0.05. For the purpose of DEG consolidation across the four datasets, R software (version 3.6.3; https://www.r-project.org/), in conjunction with the ggplot2 packages (version 3.3.3), was judiciously employed for analysis and visualization.

### 2.3. Functional and Pathway Enrichment Analysis

The enrichment of GO and KEGG pathways pertaining to both upregulated and downregulated DEGs was conducted utilizing R software (version 3.6.3; https://www.r-project.org/) and the clusterProfiler packages (version 3.14.3) [13]. Statistical significance was ascribed when *P* < 0.05.

### 2.4. PPI Network Construction

The assembly of the PPI network was accomplished through the utilization of the search tool for the retrieval of interacting genes/proteins (http://string-db.org) [14]. Subsequently, Cytoscape software (version 3.7.0; www.cytoscape.org) was aptly harnessed for both network generation and in-depth analysis, which encompassed node file scrutiny.

### 2.5. Cell Culture and Adipogenic Differentiation

The hBMSCs were procured from Cyagen, China. At passage 3, these cells were seeded into a 12-well plate at a density of 1.5 × 10^5^ cells per well and cultured in Dulbecco's modified Eagle's medium (DMEM, Gibco). The culture medium was supplemented with 10% fetal bovine serum, 100 U/mL penicillin, and 100 U/mL streptomycin. The cells were maintained at 37°C in an environment with 5% CO_2_, and the medium was refreshed every 3 days.

### 2.6. Adipogenic Differentiation and Oil Red O Staining

To induce adipogenic differentiation, the hBMSCs were cultured for 14 days in DMEM supplemented with 1 *μ*M dexamethasone, 200 *μ*M indomethacin, 0.5 mM 3-isobutyl-1-methylxanthine, 10 *μ*g/mL insulin, 10% FBS, 100 U/mL penicillin, and 100 U/mL streptomycin. Following this culture period, the cells were stained using oil red O (Sigma). All experiments were conducted in triplicate. Observation and imaging of the cells were performed using an inverted phase-contrast microscope (Nikon, Japan).

### 2.7. Total RNA Extraction and Real-Time Quantitative Polymerase Chain Reaction (RT-qPCR)

The total RNA was isolated from the hBMSCs using RNAiso Plus (Takara, Japan). Subsequently, the quality and concentration of the extracted total RNA were assessed using the Q5000 UV–Vis Spectrophotometer (Quawell, USA). Reverse transcription was carried out employing the Evo M-MLV RT Kit (AG, China). The resulting cDNA, along with specific forward and reverse primers and SYBR (Hieff® qPCR SYBR Green Master Mix, Yeasen, China), were utilized for RT-qPCR analysis.

The cycle threshold (*Ct*) values corresponding to the genes of interest were normalized against the *Ct* values of GAPDH. Relative mRNA expression levels were determined using the 2^(−*ΔΔ*CT)^ method. Detailed primer sequences can be found in Table S1.

### 2.8. MiRNA-Gene Network and TF-Gene Network Construction

The creation of the miRNA-gene network involved meticulous analysis, facilitated by miRTarBase (version 8.0; https://mirtarbase.cuhk.edu.cn/) within the Networkanalyst framework. Furthermore, the TF-gene network was meticulously curated by leveraging the ENCODE database (http://cistrome.org/BETA/) also within the Networkanalyst environment. Visual representation of these networks was meticulously executed via Cytoscape software. Notably, the cytoHubba by degree algorithm was employed to discern the top 10 miRNAs and TFs of paramount significance within the networks.

## 3. Results

### 3.1. Detection of DEGs during MSC Adipogenesis

**Figure 1:**
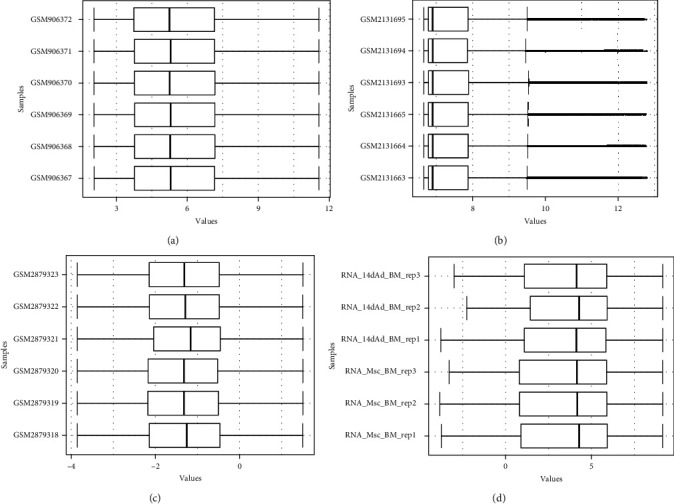
Box plots of GSE36923 (a), GSE80614 (b), GSE107789 (c), and GSE113253 (d) after normalization. The vertical axis is the name of the samples, while the horizontal axis stands for the values of expression. The black line stands for the median of data and represents the normalization degree. After normalization, the black line in each group was almost collinear, which indicates an excellent degree of normalization.

**Figure 2:**
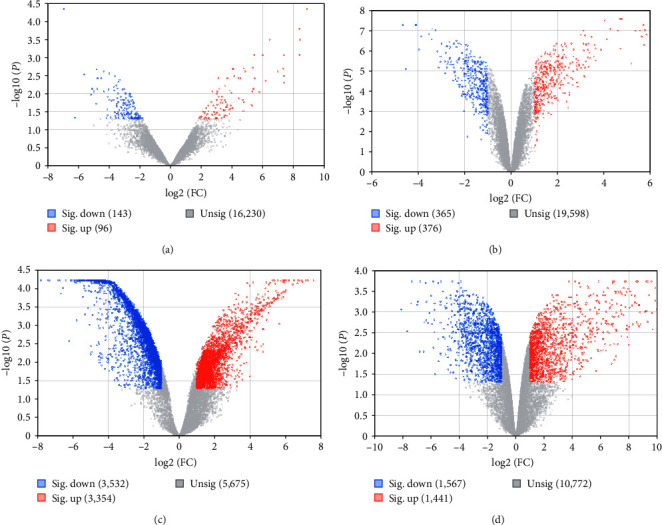
Volcano plots of differentially expressed genes of GSE36923 (a), GSE80614 (b), GSE107789 (c), and GSE113253 (d). The abscissa is log2 (FC), and the ordinates are −log10 (*P*-value). The red dots stand for the upexpressed genes, and the blue dots stand for the downexpressed genes, while the gray dots represent genes not differentially expressed.

**Figure 3:**
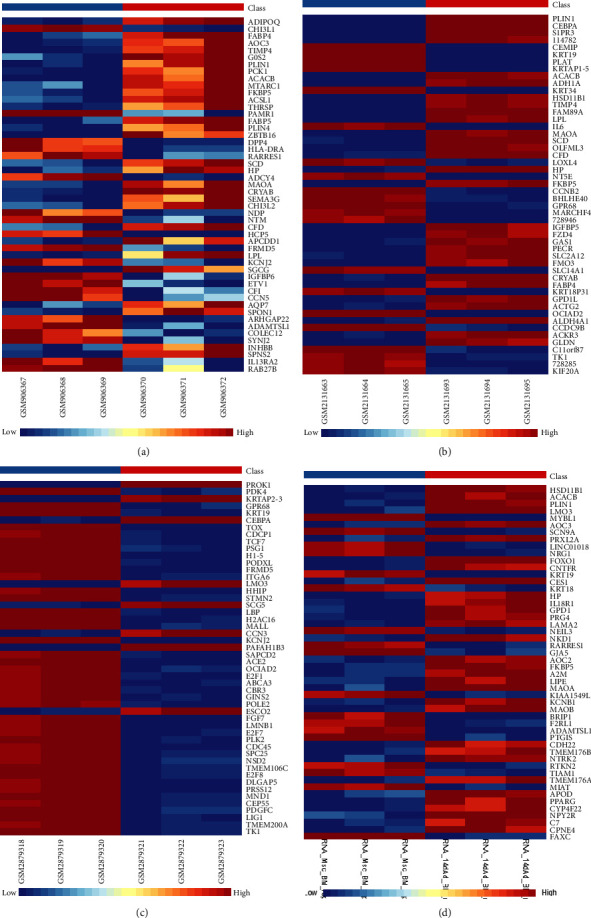
Heat maps of the differentially expressed genes (top 50 upregulated and downregulated genes) of GSE36923 (a), GSE80614 (b), GSE107789 (c), and GSE113253 (d). Red represents a high expression, and the deeper the red color, a higher expression value. Blue represents low expression, and a deeper blue color, a lower expression value.

**Figure 4:**
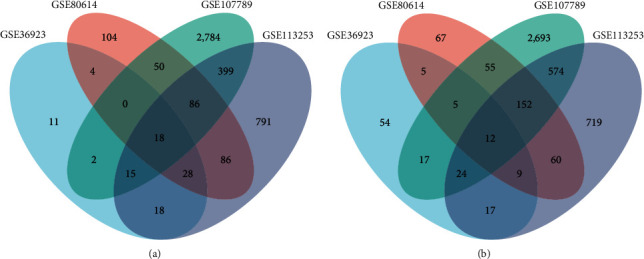
The Venn diagram of overlapped upregulated (a) and downregulated (b) differentially expressed genes of four datasets.

Following meticulous data normalization (as illustrated in Figure 1), we successfully identified a total of 239 DEGs, comprising 96 upregulated and 143 downregulated genes in the GSE36923 dataset. Similarly, in the GSE80614 dataset, a total of 741 DEGs were discerned, encompassing 376 upregulated and 365 downregulated genes. In the GSE107789 dataset, a more extensive repertoire of DEGs emerged, with 6,886 genes exhibiting differential expression—3,354 being upregulated and 3,532 downregulated. Lastly, within the GSE113253 dataset, a sum of 3,008 DEGs was unveiled, comprising 1,441 upregulated and 1,567 downregulated genes. The DEGs are artistically presented in the form of volcano plots, as exemplified in Figure 2, and the top 50 upregulated and downregulated DEGs are meticulously depicted in the heatmaps, as demonstrated in Figure 3. Remarkably, a noteworthy subset of 18 upregulated DEGs (Figure 4(a)) and 12 downregulated DEGs (Figure 4(b)) exhibited convergence across all four datasets.

### 3.2. GO Enrichment and KEGG Pathway Analysis

**Figure 5:**
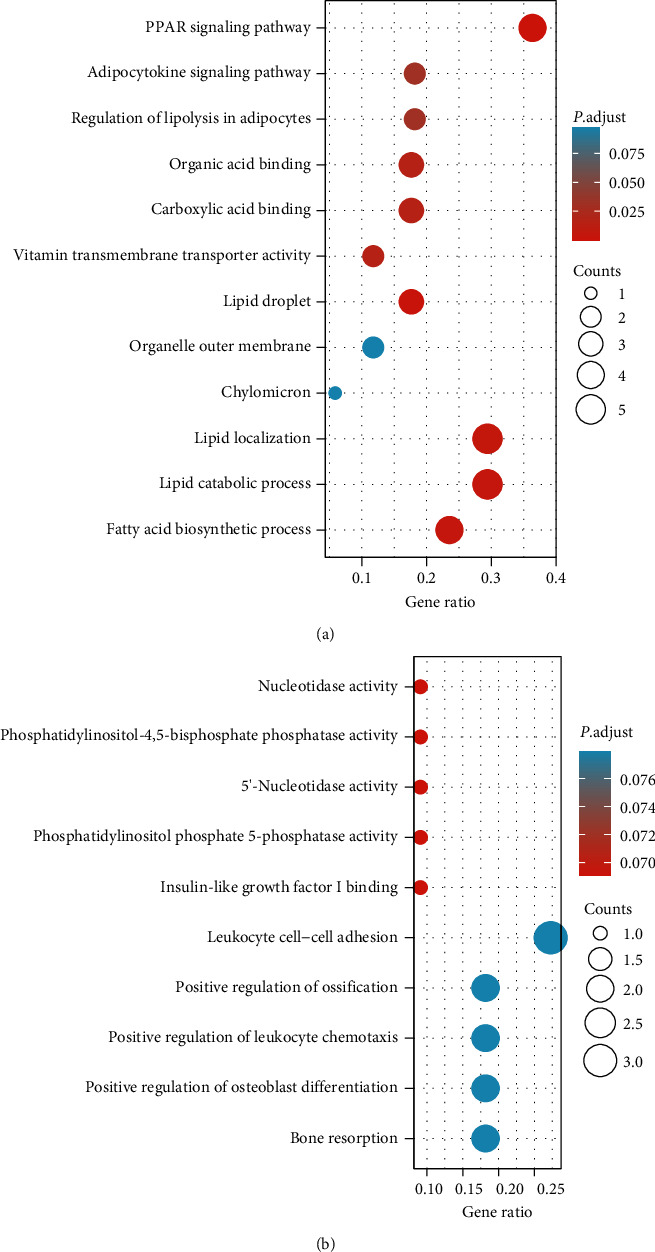
Gene ontology (GO) enrichment results and Kyoto Encyclopaedia of Genes and Genomes (KEGG) pathway analysis of overlapped upregulated (a) and downregulated (b) differentially expressed genes (DEGs). Bubble charts show the enrichment of DEGs in GO results and signaling pathways. The *x*-axis label represents the gene ratio, and the *y*-axis label represents GO terms. The size of the circle stands for gene count, and the color of the circle stands for adjusted *P* value.

A comprehensive functional enrichment analysis was systematically executed on both upregulated and downregulated genes to elucidate the functional attributes inherent to the intersecting DEGs. In the realm of molecular functions, upregulated DEGs (Figure 5(a)) were notably enriched in activities related to vitamin transmembrane transport, carboxylic acid binding, and organic acid-binding. Conversely, downregulated DEGs (Figure 5(b)) exhibited enrichment in functions encompassing insulin-like growth factor I binding, phosphatidylinositol phosphate 5-phosphatase activity, 5′-nucleotidase activity, phosphatidylinositol-4,5-bisphosphate phosphatase activity, and nucleotidase activity.

In the context of biological processes (Figure 5(a)), upregulated DEGs were principally associated with lipid catabolism, fatty acid biosynthesis, and lipid localization. On the other hand, downregulated DEGs (Figure 5(b)) were implicated in processes involving bone resorption, positive regulation of osteoblast differentiation, leukocyte cell–cell adhesion, positive regulation of leukocyte chemotaxis, and positive regulation of ossification. Pertinently, pivotal pathways, including the peroxisome proliferator-activated receptor (PPAR) signaling pathway, regulation of lipolysis in adipocytes, and the adipocytokine signaling pathway were among the top pathways implicated by DEGs (Figure 5(a)).

### 3.3. Construction of PPI Network

**Figure 6:**
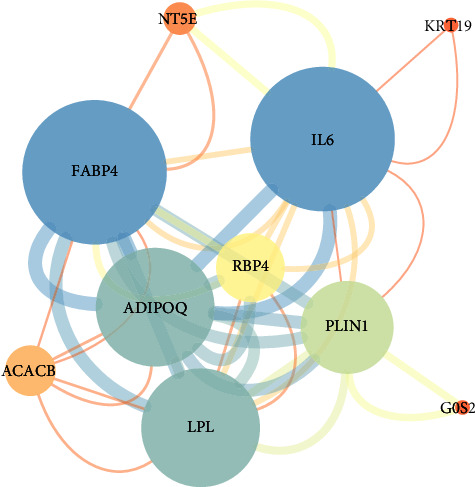
Protein–protein interaction network. The circle represents genes, and the line indicates the interactions among genes. A thicker line stands for a higher edge confidence, and a larger node size stands for a higher degree.

The construction of the PPI network, facilitated by the STRING database and executed using Cytoscape software (as portrayed in Figure 6), revealed a network comprising 10 nodes interconnected by 42 edges. These nodes corresponded to the following genes: IL6, FABP4, ADIPOQ, LPL, PLIN1, RBP4, ACACB, NT5E, KRT19, and G0S2, as summarized in Table 2.

### 3.4. Validation of mRNA Expression of 10-Node Genes during the Adipogenic Differentiation Process of hBMSCs

**Figure 7:**
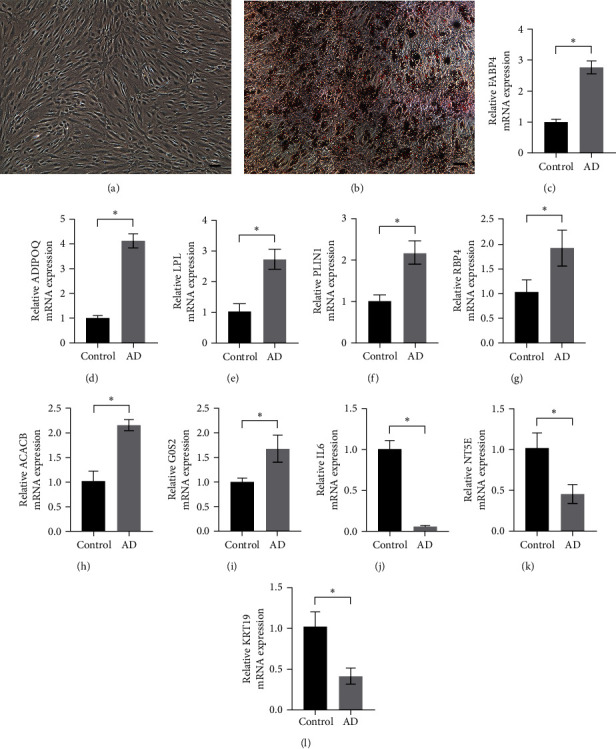
Oil red O staining and validation of 10-node genes using real-time quantitative polymerase chain reaction. The staining with Oil red O shows a comparison between adipogenic human bone marrow-derived mesenchymal stem cells (b) and controls (a). The expression levels of FABP4, ADIPOQ, LPL, PLIN1, RBP4, ACACB, and G0S2 were significantly increased (c–i), while the expression levels of IL6, NT5E, and KRT19 were significantly decreased (j–l) after adipogenic induction. *P*-values were calculated using the two-tailed Student's *t*-test, and statistical significance was considered when  ^*∗*^*P* < 0.05. Scale bar = 200 *μ*m.

We selected the 10-node genes and explored their expression levels during the adipogenic differentiation process of hBMSCs. Our findings indicate that, among the upregulated genes, FABP4, ADIPOQ, LPL, PLIN1, RBP4, ACACB, and G0S2 exhibited consistent expression patterns with the sequencing data. Additionally, among the downregulated genes, IL6, NT5E, and KRT19 showed consistency with the expression profiles observed in the sequencing data (Figure 7).

### 3.5. Establishment of miRNA–Gene Interaction Network

**Figure 8:**
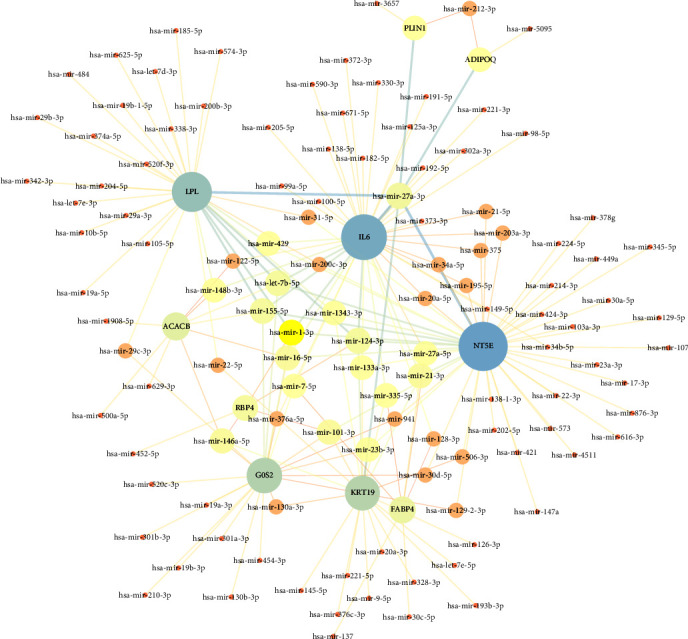
The miRNA–target gene interaction network. The circle represents genes, and the line indicates the interactions among genes. A thicker line stands for a higher edge betweenness, and a larger node size stands for a higher degree.

Exploration of miRNA–gene interactions involved the identification of 118 miRNAs targeting the 10 aforementioned node genes, achieved through the utilization of miRTarBase (version 8.0) within NetworkAnalyst. The resulting miRNA–gene interaction network, presented in Figure 8, was meticulously generated using Cytoscape. Notably, the top 10 key miRNAs, identified based on their degree centrality within the network, encompassed hsa-mir-27a-3p, hsa-let-7b-5p, hsa-mir-1-3p, hsa-mir-124-3p, hsa-mir-155-5p, hsa-mir-16-5p, hsa-mir-101-3p, hsa-mir-21-3p, hsa-mir-146a-5p, and hsa-mir-148b-3p (Table 3).

### 3.6. Establishment of TF–Gene Interaction Network

**Figure 9:**
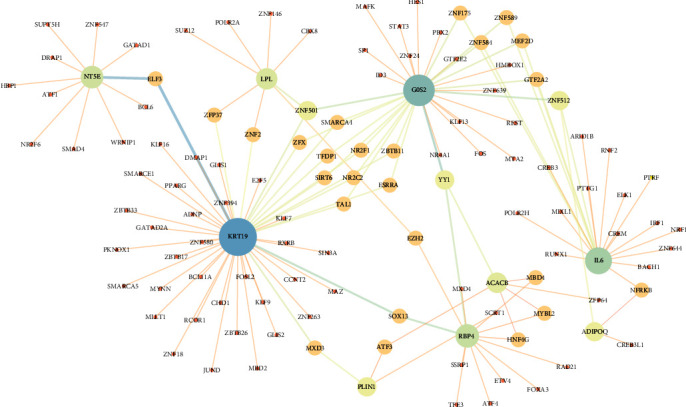
The TF–target gene interaction network. The circle represents genes, and the line indicates the interactions among genes. A thicker line stands for a higher edge betweenness, and a larger node size stands for a higher degree.

The comprehensive elucidation of TF–gene interactions involved the identification of 113 TFs targeting the 10 pivotal hub genes, accomplished through the utilization of the ENCODE software package within NetworkAnalyst. The ensuing TF–gene interaction network, artistically depicted in Figure 9 via Cytoscape, shed light on the interplay between these key regulatory elements. Notably, the top 10 crucial TFs, distinguished by their degree centrality within the network, encompassed ZNF501, ZNF512, YY1, EZH2, ZFP37, ZNF2, SOX13, MXD3, ELF3, and TFDP1 (Table 4).

## 4. Discussion

MSCs have garnered substantial attention in the field of tissue engineering due to their remarkable potential. These versatile cells find applications in the restoration of extensive soft tissue defects, which often result from traumatic injuries, surgical tumor resections, and congenital anomalies, owing to their remarkable adipogenic capabilities [15]. However, it is imperative to acknowledge the current limitations in the efficiency of adipogenic differentiation exhibited by MSCs [16]. Furthermore, the intricate link between MSC adipogenesis and prevalent health conditions, including obesity, metabolic syndrome, type 2 diabetes mellitus, and osteoporosis, underscores the need for a comprehensive understanding of the underlying mechanisms. Such insights could not only enhance the effectiveness of MSC adipogenic differentiation in tissue engineering but also pave the way for innovative therapeutic strategies targeting these diseases [17].

In a previous study, we delved into the roles of hub genes, miRNAs, and TFs in MSC adipogenic differentiation and dedifferentiation [18]. This current study extends our investigation by incorporating additional datasets and honing in on the DEGs associated with adipogenically differentiated MSCs at distinct time points: days 4, 7, and 14, extracted from four datasets. This comprehensive approach spans both early and late stages of adipogenic differentiation, acknowledging that genes participating throughout this process may exert crucial regulatory control. Remarkably, our analysis identified 18 upregulated DEGs and 12 downregulated DEGs that intersected across the four datasets.

To shed light on the functional relevance of these DEGs, we systematically examined their interactions through GO and KEGG pathway analyses. The upregulated DEGs were prominently associated with processes such as lipid catabolism, fatty acid biosynthesis, and lipid localization. Notably, pathway enrichment analysis highlighted the significance of the PPAR signaling pathway, regulation of lipolysis in adipocytes, and adipocytokine signaling pathway in the context of adipogenic differentiation. It is crucial to recognize that PPAR, a member of the nuclear hormone receptor family of TFs [19], plays a pivotal role in governing crucial aspects of MSC adipogenesis, encompassing fatty acid oxidation, transport, and synthesis [19, 20]. Intriguingly, PPAR signaling also intertwines with MSC osteogenic differentiation [21], exerting dual regulatory control over bone and adipose tissue formation [22]. Moreover, our analysis of downregulated DEGs unveiled biological processes featuring bone resorption and positive regulation of osteoblast differentiation, suggesting that MSC osteogenic differentiation undergoes concurrent modulation during adipogenesis. The intricate interplay between MSC adipogenesis and osteogenesis thus calls for further exploration.

The construction of a PPI network, facilitated by the STRING database and executed using Cytoscape, provided valuable insights into the regulatory landscape. This network encompassed 10 hub genes: IL6, FABP4, ADIPOQ, LPL, PLIN1, RBP4, ACACB, NT5E, KRT19, and G0S2, as detailed in Table 2. Notably, the roles of NT5E and KRT19 in adipogenesis warrant further investigation, as their specific contributions remain uncharted territory. The role of IL6 in MSC adipogenic differentiation remains a subject of inquiry, with studies presenting conflicting evidence, some suggesting a suppressive role [23], while others propose a promotive effect [24, 25, 26]. FABP4, ADIPOQ, LPL, and PLIN1 are well-recognized markers of adipogenesis [27, 28, 29, 30], with FABP4 emerging as a particularly promising molecular marker for MSC adipogenesis [31]. Furthermore, ACACB, implicated in lipid metabolism [32], exhibits heightened expression during bisphenol A-induced adipogenesis [33]. RBP4, a novel adipocytokine associated with obesity, also demonstrates increased expression during adipocyte differentiation [32]. Additionally, G0S2, identified as a novel target gene of peroxisome-proliferator-activated receptors [34], demonstrated a substantial rise in levels during both in vitro and in vivo adipogenesis [35, 36]. These findings collectively underscore the importance of these hub genes in orchestrating the intricate process of MSC adipogenesis, shedding light on their potential roles in this complex regulatory network.

Within our comprehensive analysis of hub miRNAs, a compelling discovery emerged—specifically, the dearth of existing reports elucidating the interactions between adipogenesis and three critical hub miRNAs: hsa-mir-101-3p, hsa-mir-21-3p, and hsa-mir-148b-3p. The uncharted territory surrounding these miRNAs piqued our scientific curiosity and beckoned for further exploration. In the realm of miRNA-mediated regulation of adipogenesis, miR-27a-3p came to the fore as a prominent figure. Shi et al. [37] underscored the substantial influence of miR-27a-3p in driving the adipogenic process. Wu et al. [38] also illuminated the inhibitory capabilities of miR-27-3p in adipogenesis. Shen et al. [39] discovered that miR-27a-3p acts an inhibitory effect on the PPAR*γ* gene to inhibit adipogenesis. Intriguingly, miR-7b-5p emerged as a regulator that exerts constraints on adipogenesis. Li et al. [40] elucidated the role of miR-7b-5p in restricting adipogenesis in MSCs. Zhi et al. [41] reported the downregulation of hsa-mir-1-3p in obese rats, while Ji et al. [42] observed its heightened expression during adipose tissue development, indicating its potential dual roles in different contexts. Pan et al. [43] highlighted the suppressive effect of miR-124-3p on adipogenesis, achieved through its targeting of C/EBP*α*. Contrarily, Liu et al. [44] proposed that myostatin utilizes miR-124-3p to inhibit adipogenesis by activating the glucocorticoid receptor. Intriguingly, miR-155 exhibited multifaceted roles in adipogenesis. Gaudet et al. [45] demonstrated that miR-155 owned the potential as an anti-obesity target. Conversely, Yu et al. [46] reported that enhanced production of miR-155-5p led to reduced adipogenesis and obesity. Xu et al. [47] found that miRNA-16-5p promoted adipogenesis through suppressing EPT1. The enigmatic role of miR-146a-5p in adipogenesis continues to elude our comprehension. While Tao et al. [48] reported its substantial induction during primary adipogenesis originating from brown adipose tissue differentiation, Hsieh et al. [49] found no significant alterations in the osteogenic and adipogenic capacities of MSCs with miR-146a-5p overexpression. Furthermore, Wu et al. [50] proposed that miR-146a-5p could suppress TNF-*α*-induced adipogenesis in primary porcine adipocytes, and Zhang et al. [51] indicated its inhibitory potential in porcine intramuscular adipogenesis.

Within the realm of TFs, our investigation has uncovered a multitude of uncharted interactions between adipogenesis and pivotal TFs, including ZNF501, ZNF512, ZFP37, ZNF2, SOX13, ELF3, and TFDP1. These intriguing revelations shed new light on the complex regulatory network governing adipogenic processes. Liu et al. [52] expanded on this by identifying YY1 as a key TF involved in both adipogenic and osteogenic differentiation of MSCs. On the other hand, Li et al. [53] suggested that YY1 exerts a suppressive effect on adipogenesis by modulating PPARG expression in bovine preadipocytes. Han et al. [54] reported a decrease in adipogenesis attributed to YY1′s inhibition of PPAR*γ* transcriptional activity in 3T3-L1 cells. Contradictorily, Huang et al. [55] proposed that YY1 promotes adipogenesis by suppressing CHOP-10 expression. The transcriptional regulator EZH2, known for its histone methyltransferase activity, demonstrates intricate regulatory dynamics in adipogenesis. Hemming et al. [56] observed that inhibiting EZH2 activity and silencing the Ezh2 gene in human MSCs led to a decrease in adipogenesis. In contrast, Wang et al. [57] illuminated a different facet of EZH2, revealing its capacity to enhance adipogenesis by repressing Wnt genes. Yiew et al. [58] offered insight into the consequences of EZH2 suppression-promoted lipoprotein-dependent lipid accumulation. Furthermore, Wan et al. [59] uncovered the collaborative interaction of EZH2 with MacroH2A1.1, contributing to increased adipogenesis by modulating Wnt signaling. Chen et al. [60] found that the EZH2-histone deacetylase 9c axis could regulate adipogenesis and osteogenesis of MSCs. Liu et al. [61] found that circSAMD4A enhanced adipogenesis through binding to miR-138-5p and increasing EZH2 expression. Dudakovic et al. [62] found that adipogenic differentiation of MSCs was suppressed when EZH2 activity was blocked. MXD3, another pivotal TF in our exploration, plays a nuanced role in adipogenesis. Shimada et al. [63] reported that MXD3 downregulation leads to reduced adipogenesis via the repression of Cebpd. Conversely, Tsai et al. [64] unveiled MXD3′s capacity to promote lipogenesis, ultimately contributing to increased body weight.

Our study faces several limitations. First, the sample size in each dataset we incorporated was limited, necessitating the inclusion of more samples to mitigate potential false-positive results. Second, the in vitro experiments conducted in this study only verify gene expression levels. Our future steps will entail progressing to in vivo and ex vivo experiments, concentrating on gene regulation, and exploring the related molecular pathways. These endeavors aim to enhance our comprehension of the underlying mechanisms governing MSC adipogenic differentiation.

In summary, we found a total of 10 hub genes (IL6, FABP4, ADIPOQ, LPL, PLIN1, RBP4, ACACB, NT5E, KRT19, and G0S2) closely related to adipogenesis of MSCs. Moreover, interaction networks of miRNA-gene and TF-gene were established, involving 118 miRNAs and 113 TFs, respectively. Taken together, these significant findings are poised to substantially enhance our comprehension of the intricate processes underpinning MSC adipogenic differentiation, setting the stage for further advancements in the field of regenerative biology and adipogenic differentiation-related diseases.

## Figures and Tables

**Table 1:** Data source.

ID	Platform	Status	Induction time (day)
GSE36923	GPL570 (HG-U133_Plus_2) Affymetrix Human Genome U133 Plus 2.0 Array	Uninduced samples: GSM906367, GSM906368, GSM906369 Adipogenic induction samples: GSM906370, GSM906371, GSM906372	14

GSE80614	GPL6947 Illumina HumanHT-12 V3.0 expression beadchip	Uninduced samples: GSM2131663, GSM2131664, GSM2131665 Adipogenic induction samples: GSM2131693, GSM2131694, GSM2131695	4

GSE107789	GPL14550 Agilent-028004 SurePrint G3 Human GE 8x60K Microarray (Probe Name Version)	Uninduced samples: GSM2879318, GSM2879319, GSM2879320 Adipogenic induction samples: GSM2879321, GSM2879322, GSM2879323	7

GSE113253	GPL18460 Illumina HiSeq 1500 (*Homo sapiens*) GPL24676 Illumina NovaSeq 6000 (*H. sapiens*)	Uninduced samples: RNA_Msc_BM_rep1, RNA_Msc_BM_rep2, RNA_Msc_BM_rep3 Adipogenic induction samples: RNA_14dAd_BM_rep1, RNA_14dAd_BM_rep2, RNA_14dAd_BM_rep3	14

**Table 2:** Ten-node genes and their descriptions.

Gene symbol	Description	Accession number	Regulation	Chromosome	Length (bp)	Degree score
IL6	Interleukin 6	NM_000600.5	Down	7	1,127	7
FABP4	Fatty acid binding protein 4	NM_001442	Up	8	911	7
ADIPOQ	Adiponectin, C1Q, and collagen domain containing	NM_001177800.2	Up	3	4,593	6
LPL	Lipoprotein lipase	NM_000237.3	Up	8	3,565	6
PLIN1	Perilipin 1	NM_002666	Up	15	2,916	5
RBP4	Retinol-binding protein 4	NM_006744	Up	10	1,070	4
ACACB	Acetyl-CoA carboxylase beta	NM_001412734	Up	12	9,855	3
NT5E	5′-nucleotidase ecto	NM_002526	Down	6	3,562	2
KRT19	Keratin 19	NM_002276	Down	17	1,390	1
G0S2	G0/G1 switch 2	NM_015714.4	Up	1	876	1

**Table 3:** Ten key miRNAs and their descriptions.

Gene symbol	Accession number	Chromosome	Length (bp)	Degree score
hsa-mir-27a-3p	NR_029501	19	78	6
hsa-let-7b-5p	NR_029479	22	83	5
hsa-mir-1-3p	NR_029662	18	85	5
hsa-mir-124-3p	NR_029670	20	87	5
hsa-mir-155-5p	NR_030784	21	65	5
hsa-mir-16-5p	NR_029525	3	81	4
hsa-mir-101-3p	NR_029836	9	79	4
hsa-mir-21-3p	NR_029493	17	72	4
hsa-mir-146a-5p	NR_029701	5	99	4
hsa-mir-148b-3p	NR_029894	12	99	3

**Table 4:** Ten key TFs and their descriptions.

Gene symbol	Description	Accession number	Chromosome	Length (bp)	Degree score
ZNF501	Zinc finger protein 501	NM_145044	3	3,060	3
ZNF512	Zinc finger protein 512	NM_032434	2	3,531	3
YY1	YY1 transcription factor	NM_003403	14	6,534	3
EZH2	Enhancer of zeste 2 polycomb repressive complex 2 subunit	NM_004456	7	2,654	2
ZFP37	ZFP37 zinc finger protein	NM_001282515	9	6,322	2
ZNF2	Zinc finger protein 2	NM_021088	2	3,580	2
SOX13	SRY-box transcription factor 13	NM_005686	1	4,076	2
MXD3	MAX dimerization protein 3	NM_031300	5	1,067	2
ELF3	E74 like ETS transcription factor 3	NM_004433	1	3,104	2
TFDP1	Transcription factor Dp-1	NM_007111	13	2,639	2

## Data Availability

The datasets supporting the conclusions of this article are available in the Gene Expression Omnibus repository, https://www.ncbi.nlm.nih.gov/geo/query/acc.cgi?acc=GSE36923, https://www.ncbi.nlm.nih.gov/geo/query/acc.cgi?acc=GSE80614, https://www.ncbi.nlm.nih.gov/geo/query/acc.cgi?acc=GSE107789, and https://www.ncbi.nlm.nih.gov/geo/query/acc.cgi?acc=GSE113253.

## Abbreviations

MSCs: Mesenchymal stem cells
GEO: Gene Expression Omnibus
DEGs: Differentially expressed genes
PPI: Protein–protein interaction
BMSCs: Bone marrow derived-mesenchymal stem cells
GO: Gene ontology
KEGG: Kyoto Encyclopedia of Genes and Genomes
RT-qPCR: Real-time quantitative polymerase chain reaction
TF: Transcription factor.

## Supplementary Materials

Supplementary Materials Table S1 describes the primer information of 10-node genes in quantitative polymerase chain reaction.
