# Supplementary material for: Identification and Validation of Hub Genes and Construction of miRNA-Gene and Transcription Factor-Gene Networks in Adipogenesis of Mesenchymal Stem Cells
Source: Stem Cells Int. 2024 Aug 29;2024:5789593. doi: 10.1155/2024/5789593 (PMC11377116; doi:10.1155/2024/5789593)
Supplement: Supplementary Materials — Table S1 describes the primer information of 10-node genes in quantitative polymerase chain reaction. [file 5789593.f1.docx]

Supplementary Table S1. The primer information

| Gene name | Primer sequence (5’ to 3’) |
| --- | --- |
| IL6-F | TTCGGTCCAGTTGCCTTCTC |
| IL6-R | TCTTCTCCTGGGGGTACTGG |
| FABP4-F | AAGTAGGAGTGGGCTTTGCC |
| FABP4-R | TGCACATGTACCAGGACACC |
| ADIPOQ-F | AGGCTTTCCGGGAATCCAAG |
| ADIPOQ-R | AAAGCGAATGGGCATGTTGG |
| LPL-F | CGAGCGCTCCATTCATCTCT |
| LPL-R | CCAGATTGTTGCAGCGGTTC |
| PLIN1-F | CAAGGAAGAGTCAGCCCCTG |
| PLIN1-R | GCACGGTGTATCGAGAGAGG |
| RBP4-F | TGAGCAGCTTCCGAGTCAAG |
| RBP4-R | GTCCTCGGTGTCTGTGAAGG |
| ACACB-F | CATGAATGGCTGCCACATCG |
| ACACB-R | GAGGGGGATCTCAGGACTGT |
| NT5E-F | ATCCAGCAGTTGAAGGTCGG |
| NT5E-R | ATGGCAGTGACTTCCTGTGG |
| KRT19-F | GAAGGATGCTGAAGCCTGGT |
| KRT19-R | CTGGGCTTCAATACCGCTGA |
| G0S2-F | CCAAGGAGATGATGGCCCAG |
| G0S2-R | GCTGCACACAGTCTCCATCA |
| GAPDH-F | AAATTCCATGGCACCGTCAAGGCT |
| GAPDH-R | CTCATGGTTCACACCCATGACGAA |
